# Decreasing Aerosol Loading in the North American Monsoon Region

**DOI:** 10.3390/atmos7020024

**Published:** 2016-02-05

**Authors:** Aishwarya Raman, Avelino F. Arellano, Armin Sorooshian

**Affiliations:** 1 Department of Hydrology and Atmospheric Sciences, Tucson, AZ 85721, USA; 2 Department of Chemical and Environmental Engineering, Tucson, AZ 85721, USA

**Keywords:** aerosol variations, southwest US, multi-satellite analysis, North American monsoon

## Abstract

We examine the spatio-temporal variability of aerosol loading in the recent decade (2005–2014) over the North American Monsoon (NAM) region. Emerging patterns are characterized using aerosol optical depth (AOD) retrievals from the NASA Terra/Moderate Resolution Imaging Spectroradiometer (MODIS) instrument along with a suite of satellite retrievals of atmospheric and land-surface properties. We selected 20 aerosol hotspots and classified them into fire, anthropogenic, dust, and NAM alley clusters based on the dominant driver influencing aerosol variability. We then analyzed multivariate statistics of associated anomalies during pre-, monsoon, and post-monsoon periods. Our results show a decrease in aerosol loading for the entire NAM region, confirming previous reports of a declining AOD trend over the continental United States. This is evident during pre-monsoon and monsoon for fire and anthropogenic clusters, which are associated with a decrease in the lower and upper quartile of fire counts and carbon monoxide, respectively. The overall pattern is obfuscated in the NAM alley, especially during monsoon and post-monsoon seasons. While the NAM alley is mostly affected by monsoon precipitation, the frequent occurrence of dust storms in the area modulates this trend. We find that aerosol loading in the dust cluster is associated with observed vegetation index and has only slightly decreased in the recent decade.

## 1. Introduction

Aerosols play a critical role in global and regional climate, monsoonal circulation, hydrological cycle, air quality, and public health (e.g., [1–4]). Such is the case for aerosols in the semi-arid regions of North America. There is growing concern about aerosols in the North American monsoon (NAM) region as studies have shown (and projected) a warmer and drier southwest United States [5], leading to increased wildfire risks and occurrence of dust storms ([6–8], and references therein). Studies have also reported the ability of aerosols in modifying NAM precipitation through direct or indirect effects [9–11] and epidemiological outbreaks from pollution [12]. While there has been increased attention in recent decades directed to aerosols in the Asian monsoon region [2,3,13,14], limited studies have examined local-to-regional characteristics and trends of aerosols in the NAM region, precluding our ability to accurately predict its response to projections of changes in environmental conditions.

NAM is a notable feature in the atmospheric circulation over North America. It is characterized by a shift in the circulation pattern due to warmer land surfaces in the southwestern United States and northwestern Mexico during May and June, resulting in an upper-level anti-cyclone over western Mexico and a pronounced increase in precipitation from convective storms over these regions (including western Texas) during July and August. Although the NAM location is centered at the Sierra Madre Occidental in Mexico, its influence on monsoon precipitation extends widely into the areas of Arizona and New Mexico. In fact, NAM provides 70% of the annual precipitation in the region [15]. We refer the reader to several studies [16–18] on further details of its spatial extent and the underlying meteorological processes. For this study, we define the NAM region to include: northern Mexico, Arizona, southern California (SoCal), New Mexico, and western Texas. In terms of aerosols, the NAM region exhibits distinct spatio-temporal patterns due to diversity in aerosol sources and sinks during its pre-monsoon (PRM: May–June), monsoon (MON: July–August), and post-monsoon (POM: September–October) phases. During May and June, the warmer and drier conditions result in fires in vegetated areas of NAM. Large dust events typically occur in the desert areas (and abandoned agricultural fields) during MON as a result of mesoscale convective storms. NAM is not only characterized by natural aerosols (dust and fire) but also by anthropogenic aerosols [19,20]. This region in fact has the top five most polluted cities for particulate matter [21]. In terms of aerosol removal, NAM experiences significant rainfall events between July and August that are quite distinct across the year for this semi-arid environment.

Past studies of aerosol trends in the United States (US) have mainly focused on the entire North American continent or the eastern US (e.g., [22,23]). For example, a recent study [24] examined the decadal trend (2000–2009) of aerosol optical depth (AOD) in the US based on NASA Terra Moderate Resolution Imaging Spectroradiometer (MODIS) and from Advanced Very high Resolution Radiometer (AVHRR) aerosol retrievals along with simulations from the Goddard Chemistry Aerosol Radiation and Transport (GOCART) model as part of a global model/data trend analyses. They found a significant decrease in AOD [24], which they attributed to the reductions in anthropogenic (combustion-related) emissions. This finding is consistent with an earlier model study [25] using the Model of Atmospheric Transport and Chemistry and the Dust Entrainment and Deposition models that reported a significant decline of the AOD trend from anthropogenic aerosol sources over the period 1980–2006. This study also revealed that the long-term trends in natural aerosol sources over the US were not significant for the given time period. Thus, more local-to-regional studies are needed as competing sources can obscure the reported global/regional trend [24].

Over the southwest US, a climatology of aerosol loading in several areas across the state of Arizona using a suite of aerosol ground-based measurements (e.g., Interagency Monitoring of PROtected Visual Environments or IMPROVE), remotely-sensed aerosol products (e.g., MODIS), as well as model output from GOCART, showed a significant range in spatio-temporal variations of aerosols within the state, depending on the proximity of the measurement site to the dominant drivers (e.g., dust, rural activities, urban pollution, *etc.*) [19]. Recently, another study in this region noted that AOD has increased by 19% over Tucson, Arizona, in the last 35 years based on two sets of AOD (400–900 nm) measurements over Tucson (*i.e.*, 1975–1977 *versus* 2010–2012) [26]. They postulated that the increase might have been contributed by urbanization and the near tripling of population in the city. These studies highlight the importance of examining the aerosol variations across the southwestern US in detail, given mixtures of drivers on aerosol loading across the region and its unique environmental conditions (including complex topography) relative to the southeast US. In light of decadal records of remotely-sensed retrievals of atmospheric composition, precipitation, and land-surface properties, there is also a unique opportunity to conduct a multivariate analysis for a more comprehensive and consistent picture of aerosol trends in the region.

In this study, we focus on assessing the spatio-temporal trends in aerosol loading across the NAM region by examining several aerosol hotpots and their associated trends during pre-monsoon (PRM), monsoon (MON), and post-monsoon (POM) seasons over the recent decade. In particular, we aim to: (1) analyze the trends in MODIS AOD from 2005–2014 over areas characterized by a dominant source or sink (*i.e.*, fire, anthropogenic, dust, and a region affected by monsoon rainfall); (2) elucidate controlling factors of these trends using correlative information from Tropical Rainfall Measuring Mission (TRMM) precipitation products (on aerosol removal), MODIS vegetation index (on dust aerosols), fire products (on fire aerosols), and CO from Measurement of Pollution in The Troposphere (MOPITT) instrument (on anthropogenic aerosols).

## 2. Methodology

### 2.1. Satellite Products

All satellite datasets were downloaded through NASA GIOVANNI web portal developed by Goddard Earth Sciences Data and Information Science Center (GES DISC) [27]. These datasets are monthly averages and were regridded to 1° × 1° to match the MODIS spatial resolution. A brief description of each data is given below, together with Table 1 which summarizes relevant information about the datasets.

#### 2.1.1. MODIS

The MODIS instrument is an imaging spectroradiometer on board two polar orbiting satellites, NASA EOS/Terra (Febraury 2000—present) and NASA EOS/Aqua (June 2002—present). It provides global coverage every one to two days with an equatorial overpass time around ~10:30 am (descending) for Terra and ~1:30 pm (ascending) for Aqua. MODIS acquires data in 36 spectral bands from 0.41 to 14 μm. It has a swath of 2330 km at cross-track and 10 km at nadir [28].

##### Aerosol Optical Depth (AOD)

We use the Level 3 (gridded) MODIS 1° × 1° Collection 5.1 (M3 mean_mean) AOD retrievals at 550 nm for both Terra and Aqua (MOD08_M3_V051) ([28] and references therein; [29,30]) to investigate trends of aerosol loading across the period 2005–2014. The Level 2 (swath) version of these retrievals are produced using three spectral wavelengths (470, 650, 2100 nm) and two algorithms (“dark target” for dark/vegetated surfaces and “deep blue” for bright surfaces such as the desert). The main aerosol product at 550 nm is derived by matching the reflectance values at different channels to the atmospheric properties using a look-up table. These retrievals have been widely used in the studies pertinent to the spatio-temporal variability of aerosols ([31,32] and references therein). We note, however, that evaluation against AERONET *in-situ* measurements of Collection 5.1 AOD products as derived using the “dark target” algorithm show overestimation of AOD [29,30]. We use the dark target for this study since the Collection 5.1 AOD retrievals using the “deep blue” algorithm were not available after December 2007 due to polarization issues in the sensor [33]. Our initial comparison of AOD between “deep blue” (from Collection 6) and “dark target” over the NAM region shows similarity in spatio-temporal patterns with “deep blue” AOD values (on average) being less by 0.1. We further note that previous studies report a shift from high to low bias of AOD from the Terra satellite after 2004 due to the degradation of Terra/MODIS’s optical response [29]. Further information about these products is discussed in detail in [29,34].

##### Fire

We use Terra/MODIS monthly mean and cloud-corrected Fire Radiative Power (FRP in units of million watts) along with MODIS fire counts (FC, counts m^−2^ day^−1^) at 1° × 1° resolution (MODIS Active Fire Product version 005 MOD14CM1) to indicate fires in the region. MODIS identifies a candidate pixel as being affected by fires if the 4 μm brightness temperature and the difference between 4 μm and 11 μm brightness temperature depart substantially from non-fire pixels. The estimated accuracy of FRP from MODIS is 15% [35,36].

##### Normalized Difference Vegetation Index (NDVI)

Aerosol loading in regions dominated by dust aerosols highly depend on the landcover characteristics (e.g., large-scale vegetation reduces dust lofting) (e.g., [37]). We use the information from NDVI to indicate potential dust mobilization in the region. MODIS/Terra provides cloud-free composites of NDVI obtained using corrected surface reflectances in visible and near infrared channels. Here, we use MODIS/Terra Level 3 monthly mean NDVI at 0.05 degree (MOD13C2) [38]. This product is based on the spatial and temporal averages of 16 day 1 km NDVI retrievals.

#### 2.1.2. Ozone Monitoring Instrument (OMI) Ultraviolet Aerosol Index (UV AI)

OMI, which is on board the NASA EOS-Aura satellite, uses backscattered UV radiation measured at two wavelengths (354 and 388 nm) to derive UV AI. OMI has a local equator crossing time at ~1:45 pm. OMI has an advantage of being more sensitive to atmospheric aerosol loading since the reflectance of most terrestrial surfaces are low at UV wavelengths [39]. We use the Level 2G OMI UV AI retrievals at 354 nm mainly to identify aerosol hotspots in the region and partly to supplement MODIS AOD for aerosol loading trend analysis. The parent resolution of the product is 0.25° × 0.25°. Due to its higher spatial resolution compared to MODIS, OMI AI is more useful in identifying localized high aerosol loading across our domain. Also, OMI UV AI is more sensitive to smoke and dust compared to MODIS AOD.

#### 2.1.3. Tropical Rainfall Measuring Mission (TRMM) Precipitation Rate

We use the monthly mean gridded “best estimate precipitation rate” (BEPR) product obtained from TRMM multi-satellite precpitation analyses (3B43 Version 7) to understand the potential impact of rainfall on the aerosol loading in the NAM region. TRMM best estimate precipitation rate dataset is a combination of data from rain gauge stations and satellite sensors providing precipitation estimates [40]. The spatial resolution of this product is 0.25° × 0.25°.

#### 2.1.4. Measurements of Pollution in the Troposphere (MOPITT) Carbon Monoxide (CO)

The MOPITT instrument is a gas correlation radiometer also onboard the NASA EOS/Terra satellite. We use MOPITT Level 3 gridded (1° × 1°) monthly total column multispectral (2.3 and 4.7 μm) CO retrievals [41,42] to indicate fire and/or anthropogenic aerosols in the region. As a product of incomplete combustion, CO is a useful tracer of fire and anthropogenic pollution. We can therefore distinguish between fire and anthropogenic aerosol signatures using the combination of MOPITT and MODIS fire products. This is further facilitated by enhanced sensitivity of MOPITT CO multispectral retrievals (thermal+near-infrared) to CO in the lowermost troposphere [41].

### 2.2. Ancillary Datasets

**Figure 1:**
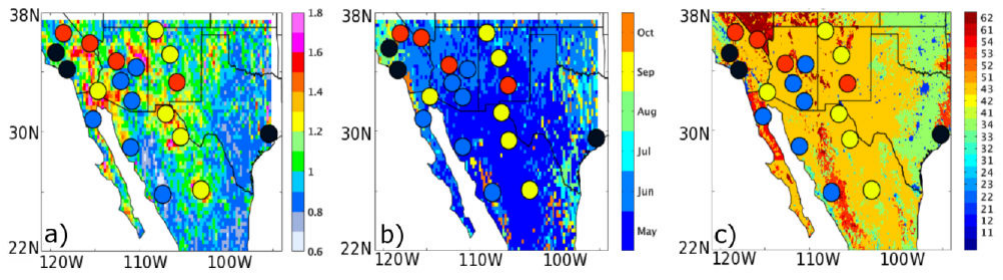
Maps of hotspot regions and aerosol clusters in NAM region. The three panels represent (**a**) the OMI UV AI climatological maximum across 2005–2014; (**b**) the month of climatological maximum; and (**c**) the anthropogenic biomes where different numbers on the colorbar indicate biome categories (11–12 are urban, 21–26 are villages, 31–35 are croplands, 41–43 are rangelands, 51–52 are forests, and 61–63 are considered wildlands). Superimposed on all three plots are the locations of 20 hotspot regions (colored circles) with the color fill indicating the aerosol cluster they belong to (red: fire, yellow: dust, blue: NAM alley, and blac anthro.

We use data on population density from Global Rural-Urban Mapping project version 1 (GRUMP V1 2000), which is provided by Socio Economic Data and Application Center (SEDAC) [43,44], to identify populated areas in the region. The data is available at 30 arc-second resolution. Along with GRUMP, we also use the anthropogenic biomes version 2 for year 2000 (also downloaded from SEDAC) to identify urban and rural areas in the region. Anthropogenic biomes or anthromes provide useful information on the alterations to the global ecosystem and biotic communities by human population [45,46]. The anthrome product has 21 anthropogenic biomes with the following six major classes: dense settlements, villages, croplands, rangelands, forested and wildlands (see Figure 1C for the different anthromes in NAM).

### 2.3. Data Analysis

#### Aerosol hotspots and clusters

We have selected a region of study bounded between the coordinates (120.5°W, 95.5°W) and (22.5°N, 37.5°N). We use the monthly decadal mean from OMI AI, along with population density and anthromes, to identify 20 aerosol hotspots in the NAM region. First, we find the climatological maximum in OMI UV AI across all six months (May to October) for each grid cell in the NAM domain. The corresponding month at which the maximum occurs for a grid cell is also identified (see Figure 1a,b). Second, we determine if a grid cell is an aerosol hotspot based on the following criteria: (1) grid value of OMI UV AI is greater than a high aerosol loading threshold value set here as AI of 1.0; (2) population density is higher than the neighboring grid cells; (3) grid cell is unique in terms of anthropogenic biome classification, topography, and/or meteorology (see Table 2). For example, although a lot of grid cells in the northwestern Mexico area satisfy the first criteria, we chose only those high aerosol cells which (a) may be affected by monsoon; (b) represent a relatively populated area that can be impacted by high aerosol loading; and (c) have distinct topographic features or anthrome classification. Our goal is to find aerosol hotspots representative of different aerosol environments. OMI UV AI is particularly useful for this purpose as it can locate smaller areas with high aerosol loading that cannot be easily detected using MODIS AOD. We note also that OMI UV AI has higher sensitivity to absorbing aerosols (e.g., black carbon and dust). Once a hotspot is identified, we re-grid OMI AI (using simple averaging) to match the 1° × 1° resolution of the MODIS AOD that we are using for this study. We define the hotspot region to include the nearest grid cells surrounding the hotspot grid cell that has climatological maximum within ±2 standard deviations of the value of the hotspot grid cell. Finally, we group the hotspot regions into four main aerosol clusters, where each cluster exhibits a distinct characteristic of aerosol variability within the NAM region. Our four clusters include: dust, NAM alley, fire, and anthropogenic (see Figure 1 and Table 2 for locations of these hotspot regions).

#### Anomalies

We start our analysis by first calculating the decadal monthly mean across 2005–2014 and the corresponding monthly standardized anomalies for each year of OMI UV AI, MODIS AOD, MODIS fire counts and FRP, MODIS NDVI, MOPITT CO, and TRMM precipitation rate. These are calculated as follows:

(1) $${\bar{x}}_{m,n}=\frac{1}{N}\sum_{y=1}^{N}\left(x_{m,n,y}\right)$$

(2) $$\sigma_{m,n}=\sqrt{\frac{\sum_{y=1}^{N}{\left(x_{m,n,y}-{\bar{x}}_{m,n}\right)}^{2}}{N-1}}$$

(3) $${x^{\prime }}_{m,n,y}=\frac{\left(x_{m,n,y}-{\bar{x}}_{m,n}\right)}{\sigma_{m,n}}$$

where (*x̄_m,n_*) is the 10-year average (*y* = 1 to *N* = 10) of a certain quantity (*x_m,n,y_*) in each 1° × 1° grid (*n*) in our domain for a particular month (*m*) (May to October) of a given year (*y*). The standardized anomaly (*x́_m,n,y_*) is calculated as the difference between (*x_m,n,y_*) and *x̄_m,n_* normalized by the 10-year standard deviation (σ*_m_,_n_*). Positive (negative) anomalies for each grid indicate values higher (lower) than its mean. We standardized the anomalies to facilitate comparison with quantities having different units (similar to [25]).

## 3. Results and Discussion

In this section, we present our results on the spatial variability in aerosol loading and decadal trends of the changes in aerosol loading over different aerosol clusters in the NAM region. The temporal trend is examined for the period 2005–2014. We express these changes as standardized anomalies to account for the variability in aerosol loading. We can interpret these anomalies to represent local rather than regional to global changes. This attempts to minimize the influence of the changes in aerosol loading due to background or transported aerosols in the region. Our analysis focuses on intra-seasonal periods corresponding to pre-monsoon, monsoon, and post-monsoon phases since the aerosol patterns vary within the monsoon season. The overall trend is further elucidated by specific trends exhibited by each cluster (fire, anthropogenic, NAM alley, and dust). We note, however, that the only aerosol dataset that shows statistically significant decadal trends in aerosol anomalies is Terra/MODIS AOD. Other aerosol datasets, such as Aqua/MODIS and Aura/OMI which both have local overpass times in the early afternoon, show considerable interannual variations of aerosol anomalies with trends (not shown) that are not statistically significant. The inability of Aqua/MODIS and Aura/OMI to exhibit significant trends can be associated with its large variability possibly due to sampling; issues, especially on observing aerosols at times when their abundance is most sensitive to cloud formation, boundary layer mixing, and convection in the region (e.g., [39]). The use of a longer period should provide sufficient samples for decadal trend analysis. For this study, however, we focus on the trends as can be inferred from Terra/MODIS.

### 3.1 Spatial Variability

**Figure 2:**
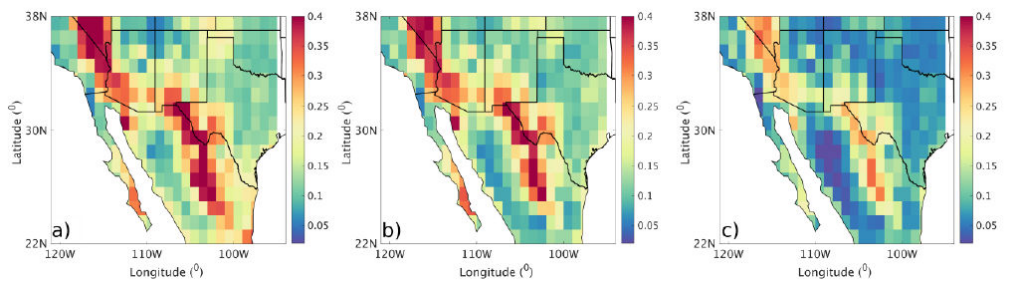
Maps of decadal mean (2005–2014) AOD from Terra-MODIS. The three panels represent (a) mean AOD during PRM; (b) MON; and (c) POM.

The decadal mean AODs (*x̄_Aod_*) for PRM, MON, and POM are shown in Figure 2. Three regions with the highest AOD patterns are apparent in these plots. The first region lies in northern California and Nevada bounded between 36°N and 38°N, and 118°W and 115°W. This region is mostly dominated by fires, especially during PRM and POM. Also, the fire cluster in this region shows the maximum mean AOD and maximum variability during PRM (Table 3). The second maxima in aerosol loading is seen in the southwestern Arizona and northern Mexico regions (30°N, 33°N, and 108°W, 41°N, 109°W). This region has been identified as an important source of dust by other studies (e.g., [19]). This region is characterized by haboob-type dust storms during MON and frontal dust storms during POM. The third source region is found between 25°N and 31°N, and 105°W and 107°W. This includes the Chihuahuan desert and Sierra Madre Oriental mountains. In all the three source regions, aerosol loading peaks in PRM and MON and decreases in POM (Table 3). In addition to these source regions, a minor region of activity, centered on LA county (34.35°N and 118.37°W), is also observed. While this region is noted for its anthropogenic pollution, recent investigations have suggested the mixing of anthropogenic and biomass burning pollution in Southern California regions (e.g., [47]).

**Figure 3:**
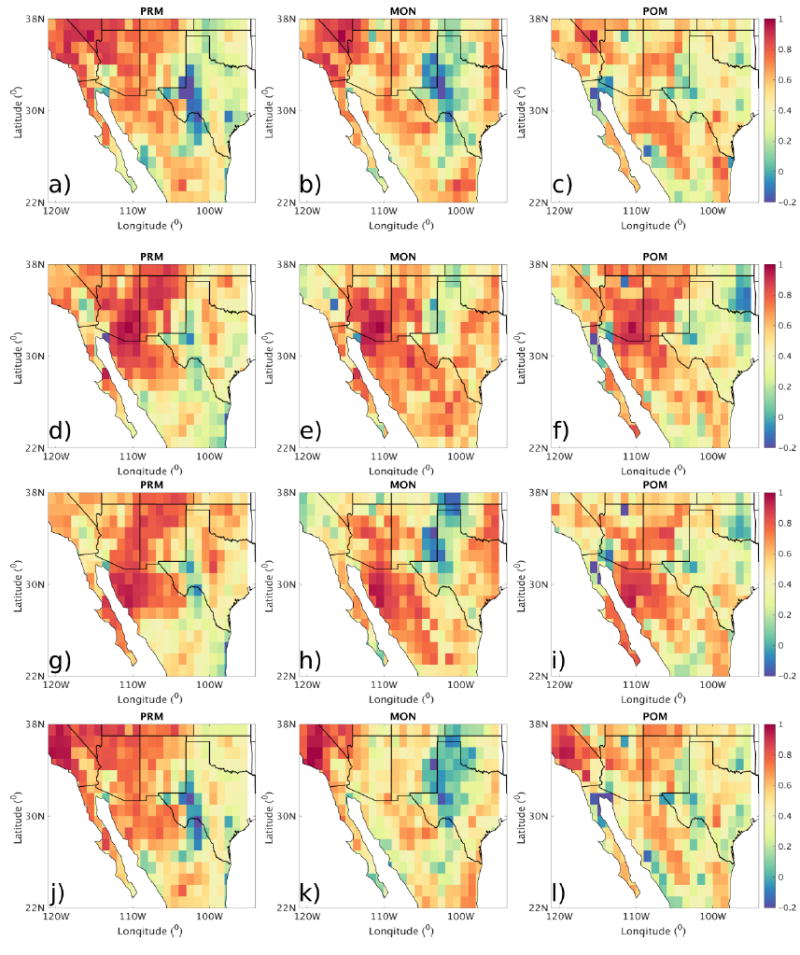
Correlation coefficient of AOD anomalies during PRM, MON, and POM. The panels (**a–c**) correspond to spatial correlation of all the grid points with respect to Charleston peak; (**d–f**) Tucson; (**g–i**) Hermosillo; (**j–l**) Bakersfield.

We also estimated the spatial correlation for these source regions by correlating AOD anomalies over a few selected places with AOD over the rest of the domain. The spatial patterns in correlation coefficients vary with season and cluster (Figure 3). The places selected in these source regions are: (1) Charleston peak (fire); (2) Tucson (NAM alley); (3) Hermosillo (dust); (4) Bakersfield (anthropogenic). Plots of correlation coefficients for these places are shown in Figure 3. For example, the peak of the regions of high correlation in AOD anomalies around Charleston is highest during PRM and decreases in MON and POM. In the case of Tucson and Hermosillo, regions of high correlation extend further into Mexico during MON and the higher correlations follow the monsoon track. We notice that, in the case of Bakersfield, regions of higher correlation extend into northern California in all the seasons. This suggests the potential similarity in frequency or magnitude of sources in between these regions. We also show spatial correlation matrices between selected aerosol hotspots (identified in Figure 1) in this supplementary material (Figure S1).

### 3.2. Overall Aerosol Trend

**Figure 4:**
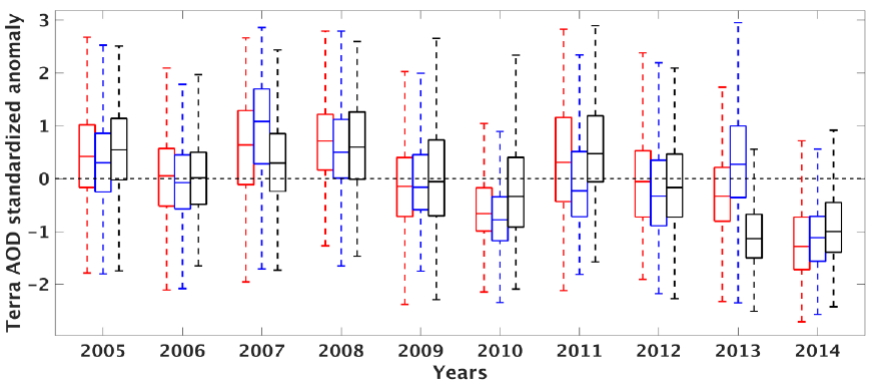
Box plots of yearly Terra/MODIS AOD standardized anomaly over the entire NAM region for the years 2005–2014. PRM (May–June), MON (July–August), and POM (September–October) periods are indicated in red, blue, and black. Whiskers represent 1.5 (IQR) above/below the upper/lower quartiles

We show in Figure 4 the yearly statistics of anomalies (*x́_aod_*) across the entire land region (region as shown in Figure 1) during PRM, MON, and POM. The statistics are summarized here using box plots where the top, central, and bottom edges of the box correspond to the 75th (*q*_3_), 50th (median), and 25th (*q*_1_) percentiles, respectively, while the whiskers correspond to ±1.5 of the interquartile range (*IQR* = *q*_3_ – *q*_1_). The interannual variability in these AOD anomalies during all seasons mostly arises from natural aerosol sources such as dust and fires. We explain this further in the following sections for different aerosol clusters. The median of the anomalies during the latter part of the decade (2010–2014) is generally negative (and lower) compared to the earlier part of the decade (2005–2009), which is generally positive (and higher) (Figure 4). This is most evident for the monsoon season. This is also the case for the interquartile range, where a shrinking of the spread in *x́_aod_* is observed in the latter part of the decade. Although there appears to be an oscillating pattern across the decade, there is a clear decreasing trend in the median and spread pointing to relatively lower aerosol loading and variability in recent years. Here, we estimate the linear trends of *x́_aod_* to be −0.14 for PRM, −0.12 for MON, and −0.15 for POM. In other words, the yearly average decrease in *x́_aod_* is greater than 10% for PRM, MON and POM, with larger decreases during PRM and POM.

### 3.3. Aerosol Trends Across Clusters

**Figure 5:**
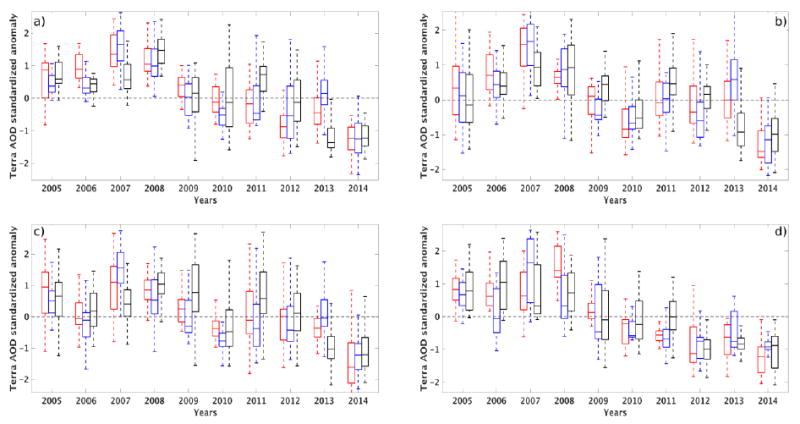
Similar to Figure 4 but for (**a**) fire; (**b**) NAM alley; (**c**) dust; and (**d**) anthro clusters.

We can further examine this decadal change by looking at the trends in the aerosel hotspot clusters. We expect more enhanced signatures of aerosol changes at these hotspot sites. In Figure 5 we show the decadal variations in *x́_aod_* within fire, dust, NAM alley, and anthro clusters. We also summarize our estimates of linear trends in Table 4. The interannual variability of *x́_aod_* is more pronounced in the fire, dust, and NAM alley, which are mostly driven by natural sources/sinks, than in the anthro cluster. This is expected as the “natural” aerosol clusters are mainly influenced by periodic atmospheric variations such as droughts and El Nino Southern Oscillation (ENSO) [48-50]. The fire and dust clusters, in particular, exhibit this periodic pattern, which can be linked to larger sources of aerosols (organic and black corbon in fires) under more arid and warmer conditions (PRM and POM). We also find a more pronounced decreaeing trend (>20%) in all clusters during PRM relative to the overall trend (>10%). However, during MON, the difference is less pronounced, except for the anthro cluster which is consistently >20% and appears to not be influenced by the monsoon . Except for the NAM alley cluster during the monsoon season, all the linear trends are significant at the 95% confidence interval (see Table 4).

The maximum decrease in *x́_aod_* can be seen in the fire cluster (27%) followed by anthro (25%). We also see a larger difference in *x́_aod_* statistics between the early and latter part of the decade in these two clusters. We see a more positive *x́_aod_* in 2005–2009 and a more negative *x́_aod_* in 2010–2014 for all three periods (PRM, MON, and POM). This decrease is most evident in the anthro cluster, which shows an increase until 2007 and then a continuous drop (both median and spread) after 2007, regardless of the monsoon season. This is consistent with the findings of [24] and [25] (albeit from a different study period) of a decreasing trend in aerosol loading which they attributed to a decreasing trend in anthropogenic aerosols. Further, a recent study on extreme events in the southwestern US also pointed out a decrease in extreme elemental carbon events between 2003 and 2013 [51], which compliments the decreasing trend in AOD anomalies in the NAM region. Our results show that the *x́_aod_* trend for the entire NAM region is mainly tied to aerosol hotspots driven mostly by anthropogenic sources that have become weaker in recent years. This is also supported (not shown here) by the decreasing trend in combustion-related aerosol emissions based on recent emission inventories (e.g., [52]) and surface measurements of particulate matter ([53]).

In the NAM alley and dust cluster, we see a very similar trend, especially during PRM. This trend, however, is lower than in the fire and anthro clusters. Differences in the trends between NAM alley and dust clusters can be seen during MON and POM, where the trend in *x́_aod_* in the dust cluster is relatively higher than in the NAM alley (see; Table 4). The oscillating pattern (as seen in [54]) is also less pronounced in the NAM alley than in the dust cluster. This difference can be attributed to an apparent modulation of *x́_aod_* in the NAM alley cluster by more frequent occurrences of convective dust storms or haboobs in this region than in the dust cluster. These storms are typically produced from isolated thunderstorms that merge to form cold pools during monsoon season. These cold pools result in severe downburst winds that lift massive quantities of dust off the surface ([8] and references therein). Based on the storm event database [55] over Arizona, we find that, although the trends in haboob-type dust storms across this study period are not statistically significant, there appears to be an increase in the frequency of dust storms over Arizona in most recent years, especially during the monsoon period. Despite the increase in rainfall (aerosol removal) during this period (which is common to both NAM alley and dust, albeit with a slight shift in timing), we infer that the increase in dust sources in the NAM alley obscures the decrease in the trend as seen in the dust cluster.

### 3.4. Multivariate Correlations

As noted earlier, we use a suite of satellite products (see Table 1) to corroborate the trends that we found in NAM and aerosol clusters. We present here the correlations between *x́_aod_* and the following anomalies across different aerosol clusters and monsoon periods: Aqua/MODIS AOD (*x́_aod___aqua_*), Aura/OMI AI (*x́_ai_*), Terra/MODIS FRP (*x́_frp_*), FC (*x́_fc_*), and NDVI (*x́_ndvi_*), Terra/MOPITT CO (*x́_CO_*), and TRMM BEPR (*x́_rain_*). These quantities provide unique first-order information on aerosol source/sink types. We note that the trends in *x́_aod_* can be influenced by several confounding factors other than those mentioned here, such as trans-Pacific transport of aerosols [56,57], mixing of aerosol emissions from within North America, removal efficiency, injection height, and frequency of emissions. Although we expect that the actual relationship between *x́_aod_* and these quantities may be nonlinear, we only examine the linear component of this relationship as a first-order approximation. More robust and quantitative assessments of aerosol trends including source attribution require modeling of aerosol sources, transport, and sinks, which is beyond the scope of this study. We note, however, that our analysis can be made useful to show observational constraints of these trends.

**Figure 6:**
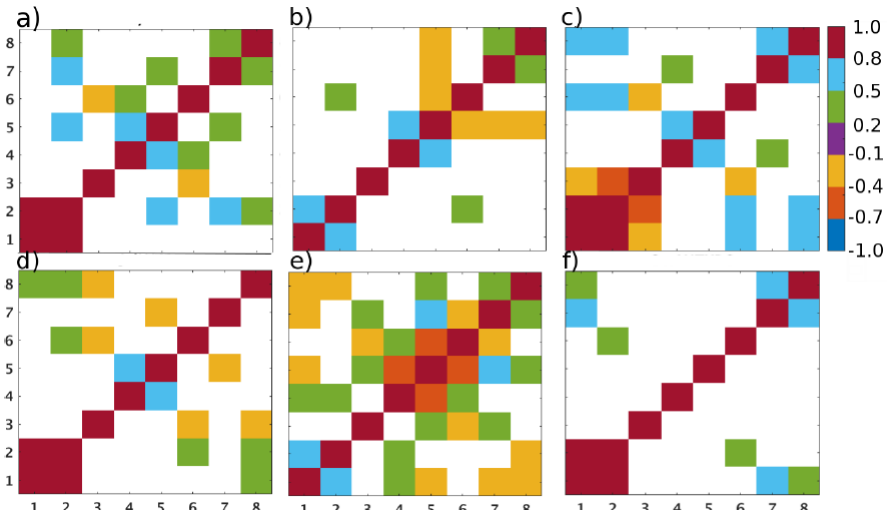
Anomaly correlation matrices between different aerosol-related quantities. The plots show correlation only during months that show significant correlation between the variables. The standardized anomalies considered here are the following: (1) Terra/MODIS AOD; (2) Aqua/MODIS AOD; (3) Aura/OMI UV AI; (4) Terra/MODIS FRP (in MW); (5) Terra/MODIS fire counts; (6) Terra/MOPITT CO (in ppbv); (7) Terra/MODIS NDVI; and (8) TRMM precipitation rate (in mm/day). Positive correlations are shown only if they are greater than 0.3 and significant at 90% confidence. Negative correlations are shown if they are significant at 90% confidence. The plots correspond to (**a**) fire cluster in May; (**b**) dust cluster in july; (**c**) anthro cluster in July; (**d**) NAM alley cluster in July; (**e**) dust cluster in September; and (**f**) anthro cluster in October.

Our results are presented in Figure 6. The correlation coefficient indicates how closely the changes in aerosol loading mirror the changes in these quantities. Here, we focus our analysis on large and significant correlations between anomalies. While high correlation between *x́_aod_* and *x́_aod_aqua_* is expected, we find a lesser correlation in the dust cluster (Figure 6b,e). This may be due to differences in retrievals of aerosols in Terra and Aqua. In the fire cluster, however, *x́_aod_aqua_* is correlated with *x́_fc_* and *x́_ndvi_* (>0.5) in May (see Figure 6a). This is also seen from the decrease in the fire counts during June for the period 2005–2014 (Figure S2). The high correlation implies that most of the anomalies in aerosol loading in this cluster are directly tied to fire aerosols. As the fuel available from biomass increases, the probability of fires also increases, especially when the region tends to get drier in the period. This is usually the case for the summer fires in California [58]. There is also high correlation between *x́_fc_* and *x́_frp_* and to a lesser extent between *x́_ai_* and *x́_co_* which may indicate higher organic (and black) carbon aerosol emissions during mixtures of intense/less intense (flaming/smoldering) fires. This is fairly consistent with the previous studies that suggest that the interannual variability in natural AOD across the US is due to the variability of organic carbon emissions from droughts and biomass burning events [24,50]. However, this result needs to be supported by surface measurements and other datasets.

During MON, correlations in the NAM alley cluster show a strong positive relationship between *x́_aod_*, *x́_rain_* and *x́_aod_aqua_* (July: Figure 6b,d) which is not apparent in the dust cluster. This implies that the anomalies in aerosol loading and precipitation rates are moving in the same direction in the NAM alley cluster during the monsoon season. Although, in general, we expect negative correlation between *x́_rain_*, and *x́_aod_*, this result suggests that the dust sources, especially from dust storms during the monsoon period, offset the aerosol removal due to rain. This can be contrasted with the dust cluster in September (Figure 6e) where there is a strong negative correlation between *x́_rain_*, *x́_ndvi_* (see Figure S3 for time series of NDVI) and *x́_aod_*, implying the stronger influence of precipitation in the absence of dust storms (and lesser vegetation) (e.g., [59,60]) and despite potentially more intense fires in this region (*i.e.,* negative correlation between *x́_co_*,*x́_fc_* and *x́_frp_*, positive correlation between *x́_aod_* and *x́_frp_*, and between *x́_frp_* and *x́_ai_* implies more intense flaming fires—less CO, high FRP at lower fire counts, and high black carbon (e.g., [61]).

In the anthro cluster during July, we find strong positive correlations between *x́_aod_*, *x́_aod_aqua_* and *x́_co_*, *x́_rain_* and a negative correlation between *x́_aod_* and *x́_ai_* (Figure 6c). Although UV AI has been noted for its sensitivity to both dust and black carbon (BC) aerosols, previous studies have also indicated that UV AI is more sensitive to dust aerosols than smoke or BC [19]. In the absence of a positive correlation with fire indicators (FRP and FC), it is evident that anomalies in aerosol loading in the anthro cluster are directly related to anthropogenic combustion. A positive correlation between *x́_rain_* and *x́_aod_* in MON (Figure 6c) and POM (Figure 6f) explains, to some extent, the lesser impact of precipitation to *x́_aod_* in the anthro cluster. This indicates that this region is mostly driven by anthropogenic (*x́_co_*) and biogenic (*x́_ndvi_*) aerosol sources than precipitation (aerosol sinks) in October.

### 3.5. AOD Sensitivity

**Figure 7:**
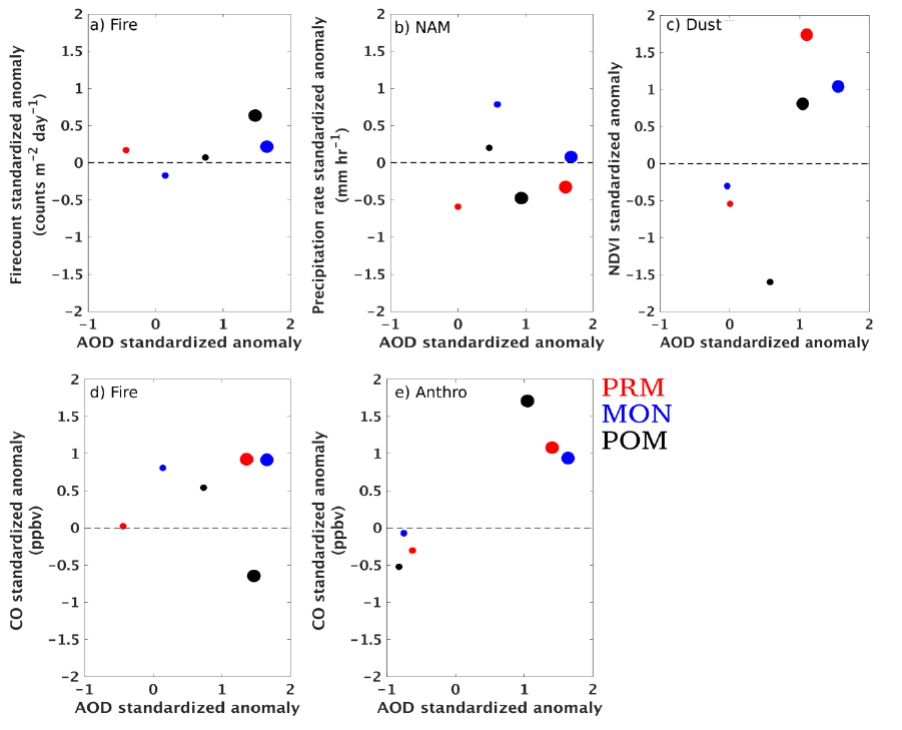
Scatterplots of standardized anomalies for selected clusters and monsoon periods. The filled circles represent local maxima values, with the larger and smaller circles corresponding to local maxima within 2005–2009 and 2010–2014, respectively. The colors represent different periods (red for PRM, blue for MON and black for POM). The different plots correspond to AOD anomalies *versus* (**a**) fire counts for fire; cluster; (**b**) precipitation for NAM alley cluster; (**c**) NDVI for dust cluster; (**d**) CO for fire cluster; and (**e**) CO for anthro cluster.

Here, we examine the difference in standardized anomalies between the maximum within the early (2005–2009) and latter parts of the decade (2010–2014). This is shown in Figure 7 for selected clusters and years with distinct sensitivities. We focus on contrasting the local maximum between these two segments of the decadal period to support the trend and correlation analysis previously discussed. Together, they provide information on the main factors of change in aerosol loading for different clusters. In Figure 7a we show the sensitivity of *x́_aod_* to *x́_fc_* in the fire cluster, suggesting a similar shift in *x́_aod_* and *x́_fc_* from positive to negative anomalies in the latter part of the decade (2010–2014) for all monsoon periods (also see Figure S2). This is consistent with the strong correlation between *x́_aod_aqua_* and *x́_fc_* shown in Figure 7. Although the largest decrease in *x́_fc_* occurs in PRM, the maximum local sensitivity (steeper slope) in *x́_fc_* with *x́_aod_* is observed during POM (*i.e.*, large change in fire counts is tied with smaller change in AOD). This reveals that AOD is more associated with smoldering fire aerosols (more emissions) during POM than in PRM. This is also indicated in Figure 7d where *x́_co_* shifts from a negative to positive anomaly during POM and shows high sensitivity with *x́_aod_*. However, the apparent decrease in fire counts shown in Figure 7a cannot be directly linked to a decrease in intensity or frequency of fires since this scatterplot is only a snapshot of this change.

During MON and POM in the NAM alley cluster, the *x́_aod_* decrease is associated with an increase in *x́_rain_* (Figure 7b and Figure S4). This sensitivity is mostly influenced by data in August (for MON) rather than July where dust storms may modulate this relationship (as we have seen in Figure 6d). Similarly we find the decrease in *x́_aod_* is associated with the decrease in *x́_ndvi_*, with the highest sensitivity during POM in the dust cluster. The small change in AOD despite a large change in NDVI points to confounding factors such as fires, aerosol transport, and atmospheric moisture during POM in this region.

Finally, in the anthro cluster, we see a consistent shift in *x́_aod_* and *x́_co_* from positive to negative anomalies across the monsoon season (Figure 7e and Figure S5). This is very consistent with the trend and correlation results for this clusler.

### 3.6. Comparison between OMI UV AI and MODIS AOD during MON

**Figure 8:**
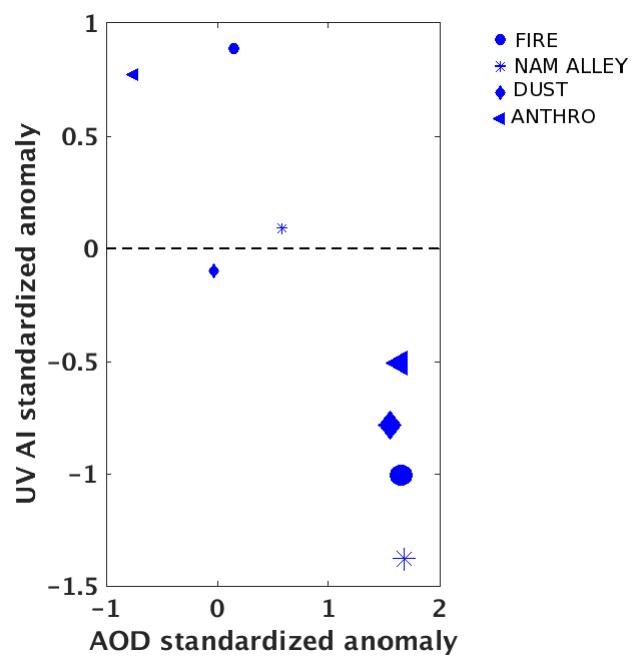
AOD *versus* UV AI standardized anomalies during the monsoon period (July–August). As in Figure 7, the larger (smaller) size symbols correspond to local maximum within 2005–2009 (2010–2014).

As noted already, the UV AI product is useful in aerosol hotspot identification because of its finer spatial resolution. Although UV AI did not show significant trends, previous studies have demonstrated the capability of UV AI in capturing smoke and dust aerosols. We present in Figure 8 the scatterplot between *x́_ai_* and *x́_aod_* during MON to demonstrate the utility of UV AI for periods exhibiting large AOD variability. The different symbols represent different clusters with the smaller sizes indicating the last local maxima value in the time segment 2010–2014, and larger sizes indicating the first local maxima values in the time segment 2005–2010. Although these two quantities cannot be directly compared in terms of magnitude (hence the standardized anomalies), we expect these variables to move in the same direction. In contrast, *x́_ai_* and *x́_aod_* show an inverse relationship during MON in all the source regions. One possible reason for the apparent increase in *x́_ai_* during MON (and POM) could again be the difference in equatorial crossing times of the satellites. Convection builds up in the morning and winds increase in intensity in the afternoon over these regions during the monsoon season. Therefore, windblown dust captured by Aura/OMI UV AI is not seen in Terra/MODIS AOD. Other potential reasons for the discrepancies between Terra/ AOD and OMI UV AI are differences in spatial resolution, sampling differences due to overpass times, retrieval characteristics and sensitivities (OMI UV AI retrieval is sensitive to black carbon and dust) (e.g., [62] and references therein). However, the results in Figure 8 show that OMI UV AI can provide additional constraints on aerosols in this region where mixed (confounding) processes are involved, making it challenging to infer aerosol trends. Future studies, based on the emission database and particulate matter concentrations, are required in the NAM region to provide a deeper understanding of the discrepancies in between these products.

## 4. Conclusions

We investigate the spatial and temporal variability of aerosol loading in the NAM region using retrievals of AOD from Terra-MODIS for the years 2005–2014 during the monsoon season (May–October). We interpret the trends in AOD using other correlative information such as NDVI, CO, rainfall rate and active fire products. The decadal average of AOD over the study area shows that the maximum aerosol loading occurs during May and June (PRM) in active source regions. Major aerosol source regions are northern California (fires), southwestern Arizona and northern Mexico (dust), and southern California (anthropogenic). Monsoonal rainfall in Arizona and Mexico acts as a major sink during this period.

We identified four aerosol clusters in the NAM region and selected 21 aerosol hotspots in the entire study area. We conducted a series of statistical analyses for these four cluster regions (fires, dust, NAM alley, and anthropogenic) and for three periods of the monsoon season (pre-, monsoon, and post-monsoon). We correlated the anomalies of AOD to fire counts and FRP (for fires), CO (for combustion), NDVI (for dust) and precipitation rates for aerosol removal) to explain the trends in aerosol loading. Our results show a significant interannual variability in AOD anomalies in fire, NAM alley and dust clusters which could be linked to climatic events. However, these speculations warrant further analyses of climatic products. The temporal trends in AOD anomaly exhibit a statistically significant decreasing trend in fire (19%–27%) and anthropogenic clusters (17%–21%). The trends in the fire cluster can be attributed to the decreasing trend in the fire counts. The decreasing trends in the NAM alley (13%–21%) and dust clusters although statistically significant, are influenced by compensating or nullifying processes such as haboobs, increased moisture during the monsoon period, rainfall, *etc.* While rainfall removes aerosol in this region, processes such as convective dust storm cause massive enhancements of dust loading.

In summary, this study highlights the need for augmenting and integrating observing systems of aerosols (and correlative measurements) in this region with high spatio-temporal resolution datasets. While anthropogenic aerosols show a clear statistically significant decreasing trend, trends in natural aerosol clusters such as dust and the NAM alley are still unclear due to the complex interplay between sources and sinks in this region. A next step would be to corroborate these results with surface observations of PM_10_ and PM_2.5_ and the emission database. We would also like to note that we have not fully considered the associated biases of the retrieval products, which is an important limitation of this study. We plan to investigate these limitations in our future work using chemical transport models and ground observations.

## Supplementary Material

Supplement Figure S1: Correlation matrices for AOD anomalies all the hotspots between the months May–October. The hotspots considered here are: (1) Mt.Whitney, CA; (2) Charleston Peak, CA; (3) Tucson, AZ; (4) Baja, CA; (5) Phoenix, AZ; (6) Phoenix, AZ; (7) Yuma, AZ; (8) LA county, CA; (9) Bakersfield, CA; (10) Prescott, AZ; (11) Petrified forest, AZ; (12) White mountain, NM; (13) Farmington, NM; (14) Albuquerque, NM; (15) Ejido El Vergel, Mex; (16) Chihuahuan Desert, Mex; (17) Hermosillo, Mex; (18) Sierra Madre Occidental, Mex; (19) Sierra Madre Oriental, Mex; (20) Houston, TX; (21) Waco, TX. The panels correspond to (A) May; (B) June; (C) July; (D) August; (E) September; (F) October.
Figure S2: Box plots of Terra/MODIS firecount standardized anomaly over the fire cluster for the years 2005–2014 between the months May–October. Whiskers represent 1.5 (IQR) above/below the upper/lower quartiles. The panels correspond to (A) May; (B) June; (C) July; (D) August; (E) September; (F) October.
Figure S3: Box plots of Terra/MODIS NDVI standardized anomaly over the dust cluster for the years 2005–2014 between the months May–October. The panels correspond to (A) May; (B) June; (C) July; (D) August; (E) September; (F) October.
Figure S4: Box plots of TRMM rainfall rate standardized anomaly over the NAM alley for the years 2005–2014 between the months May–October. The panels correspond to (A) May; (B) June; (C) July; (D) August; (E) September; (F) October.
Figure S5: Box plots of MOPITT CO standardized anomaly over the anthropogenic cluster for the years 2005–2014 between the months May–October. The panels correspond to (A) May; (B) June; (C) July; (D) August; (E) September; (F) October.

## Figures and Tables

**Table 1:** Analysis datasets.

Instrument and Dataset	Resolution	Relevance to Study (Main Product Reference)
NASA Terra and Aqua L3 MODIS Aerosol Optical Depth (AOD) 550 nm	1° × 1°	aerosol loading (Levy *et al.*, 2007 [29])

NASA Terra L3 MODIS Fire Radiative Power (FRP) and fire counts	1° × 1°	fire sources (Wooster *et al.*, 2005 [36])

NASA Terra L3 MODIS Normalized Vegetation Index (NDVI)	0.05° × 0.05°	biogenic and dust sources (Lunetta *et al.*, 2006 [38])

NASA OMI L2G UV Aerosol Index (AI) 354 nm	0.25° × 0.25°	aerosol cluster identification (Torres *et al.*, 2007 [39])

NASA MOPITT L3 TIR/NIR Total Column CO	1° × 1°	combustion sources (Deeter *et al.*, 2012 [41])

NASA TRMM Best Estimate Precipitation Rate (BEPR)	0.25° × 0.25°	aerosol removal (Huffman *et al.*, 2007 [40])

UMBC Anthropogenic Biomes V2 (2000) (ecotope.org)	0.083° × 0.083°	aerosol cluster identification (Ellis *et al.*, 2010 [45])

NASA SEDAC Global Rural Urban Mapping Project version 1 (GRUMPv1) Population Density (sedac.ciesin.columbia.edu)	1 km × 1 km	aerosol cluster identification (Balk *et al.*, 2009 [43])

**Table 2:** Hotspot sites and associated aerosol cluster information.

Site No.	Site Name	Latitude (deg N)	Longitude (deg W)	Aerosol Cluster
1	Mt. Whitney, CA	36.557	118.50	Fire
2	Charleston Peak, CA	36.29	115.69	Fire
3	Tucson, AZ	32.22	110.93	NAM alley
4	Baja, CA	30.98	115.38	NAM alley
5	Phoenix, AZ	33.45	112.08	NAM alley
6	Yuma, AZ	32.69	114.63	Dust
7	LA County, CA	34.35	118.37	Anthro
8	Bakersfield, CA	35.37	119.02	Anthro
9	Prescott, AZ	34.54	112.46	Fire
10	Petrified Forest, AZ	34.41	110.65	NAM alley
11	White Mountain, NM	33.41	105.74	Fire
12	Farmington, NM	36.73	108.22	Dust
13	Albuquerque, NM	35.01	106.61	Dust
14	Ejido El Vergel, Mex	31.20	106.59	Dust
15	Chihuahuan Desert, Mex	29.52	105.48	Dust
16	Hermosillo, Mex	29.07	110.97	NAM alley
17	Sierra Madre Occidental, Mex	25.96	107.53	NAM alley
18	Sierra Madre Oriental, Mex	26.12	103.10	Dust
19	Houston, TX	29.74	95.36	Anthro
20	Waco, TX	31.55	97.15	Dust

**Table 3:** Decadal mean and standard deviation of AOD for cluster regions.

Cluster	Season	Mean (*x̄_Aod_*)	Standard Deviation (σ*_AOD_*)
**Fire**	**PRM**	**0.23**	**0.12**
	**MON**	**0.21**	**0.10**
	POM	0.15	0.09
NAM alley	PRM	0.19	0.08
	MON	0.18	0.09
	POM	0.11	0.07
Dust	PRM	0.23	0.11
	MON	0.23	0.11
	POM	0.16	0.08
anthro	PRM	0.13	0.02
	MON	0.14	0.02
	POM	0.10	0.01

**Table 4:** Linear trends in Terra/MODIS AOD anomalies for different aerosol clusters.

Clusters	PRM (*n* = 10)	MON (*n* = 10)	POM (*n* = 10)
Fire	−0.27 (0.0002)	−0.19 (0.02)	−0.21 (0.01)
NAM alley	−0.21(0.01)	−0.15 (0.11)	−0.13 (0.06)
Dust	−0.21 (0.007)	−0.17(0.03)	−0.17(0 .02)
Anthro	−0.25 (0.0007)	−0.21 (0.009)	−0.22 (0.0001)
entire domain	−0.14 (0.02)	−0.12 (0.07)	−0.15 (0.01)

Note: values in the brackets refer to the *p*-value for the trends.
