# Supplementary material for: Decreasing Aerosol Loading in the North American Monsoon Region
Source: Atmosphere (Basel). Author manuscript; Available in PMC 2017 May 8. (PMC5422029; doi:10.3390/atmos7020024)
Supplement: Supplement — Figure S1: Correlation matrices for AOD anomalies all the hotspots between the months May–October. The hotspots considered here are: (1) Mt.Whitney, CA; (2) Charleston Peak, CA; (3) Tucson, AZ; (4) Baja, CA; (5) Phoenix, AZ; (6) Phoenix, AZ; (7) Yuma, AZ; (8) LA county, CA; (9) Bakersfield, CA; (10) Prescott, AZ; (11) Petrified forest, AZ; (12) White mountain, NM; (13) Farmington, NM; (14) Albuquerque, NM; (15) Ejido El Vergel, Mex; (16) Chihuahuan Desert, Mex; (17) Hermosillo, Mex; (18) Sierra Madre Occidental, Mex; (19) Sierra Madre Oriental, Mex; (20) Houston, TX; (21) Waco, TX. The panels correspond to (A) May; (B) June; (C) July; (D) August; (E) September; (F) October. Figure S2: Box plots of Terra/MODIS firecount standardized anomaly over the fire cluster for the years 2005–2014 between the months May–October. Whiskers represent 1.5 (IQR) above/below the upper/lower quartiles. The panels correspond to (A) May; (B) June; (C) July; (D) August; (E) September; (F) October. Figure S3: Box plots of Terra/MODIS NDVI standardized anomaly over the dust cluster for the years 2005–2014 between the months May–October. The panels correspond to (A) May; (B) June; (C) July; (D) August; (E) September; (F) October. Figure S4: Box plots of TRMM rainfall rate standardized anomaly over the NAM alley for the years 2005–2014 between the months May–October. The panels correspond to (A) May; (B) June; (C) July; (D) August; (E) September; (F) October. Figure S5: Box plots of MOPITT CO standardized anomaly over the anthropogenic cluster for the years 2005–2014 between the months May–October. The panels correspond to (A) May; (B) June; (C) July; (D) August; (E) September; (F) October. [file NIHMS822263-supplement-Supplement.pdf]

Supplementary Materials: Decreasing Aerosol Loading in the North American Monsoon Region

Aishwarya Raman, Avelino F. Arellano Jr. and Armin Sorooshian

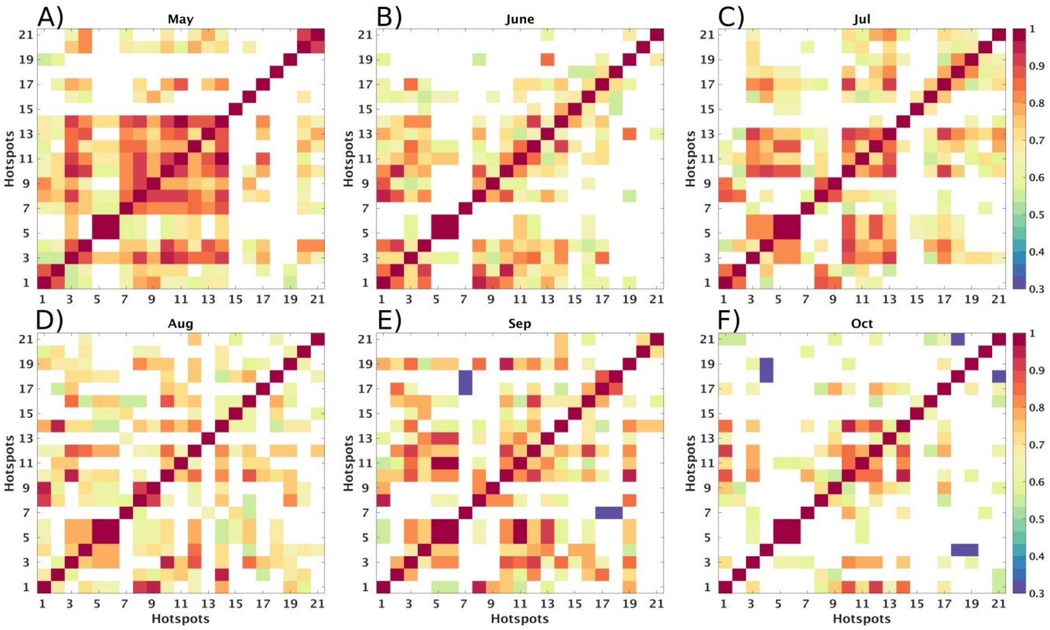

Figure S1. Correlation matrices for AOD anomalies all the hotspots between the months May–October. The hotspots considered here are; (1) Mt.Whitney, CA; (2) Charleston Peak, CA; (3) Tucson, AZ; (4) Baja, CA; (5) Phoenix, AZ; (6) Phoenix, AZ; (7) Yuma, AZ; (8) LA county, CA; (9) Bakersfield, CA; (10) Prescott, AZ; (11) Petrified forest, AZ; (12) White mountain, NM; (13) Farmington, NM; (14) Albuquerque, NM; (15) Ejido El Vergel, Mex; (16) Chihuahuan Desert, Mex; (17) Hermosillo, Mex; (18) Sierra Madre Occidental, Mex; (19) Sierra Madre Oriental, Mex; (20) Houston, TX; (21) Waco, TX. The panels correspond to (**A**) May; (**B**) June; (**C**) July; (**D**) August; (**E**) September; (**F**) October.

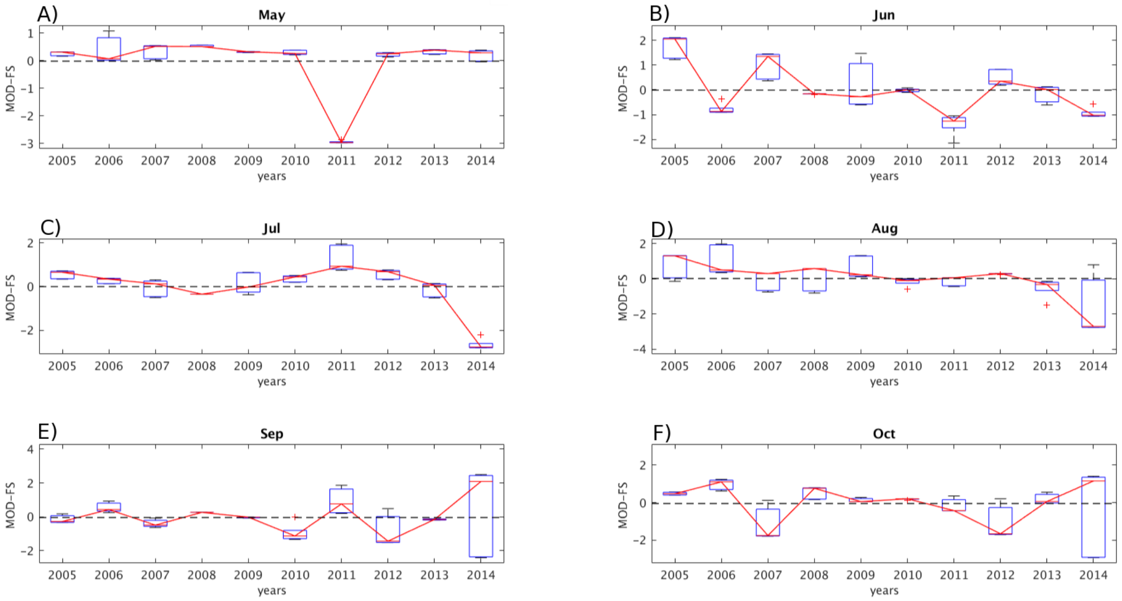

Figure S2. Box plots of Terra/MODIS firecount standardized anomaly over the fire cluster for the years 2005–2014 between the months May–October. Whiskers represent 1.5 (IQR) above/below the upper/lower quartiles. The panels correspond to (**A**) May; (**B**) June; (**C**) July; (**D**) August; (**E**) September; (**F**) October.

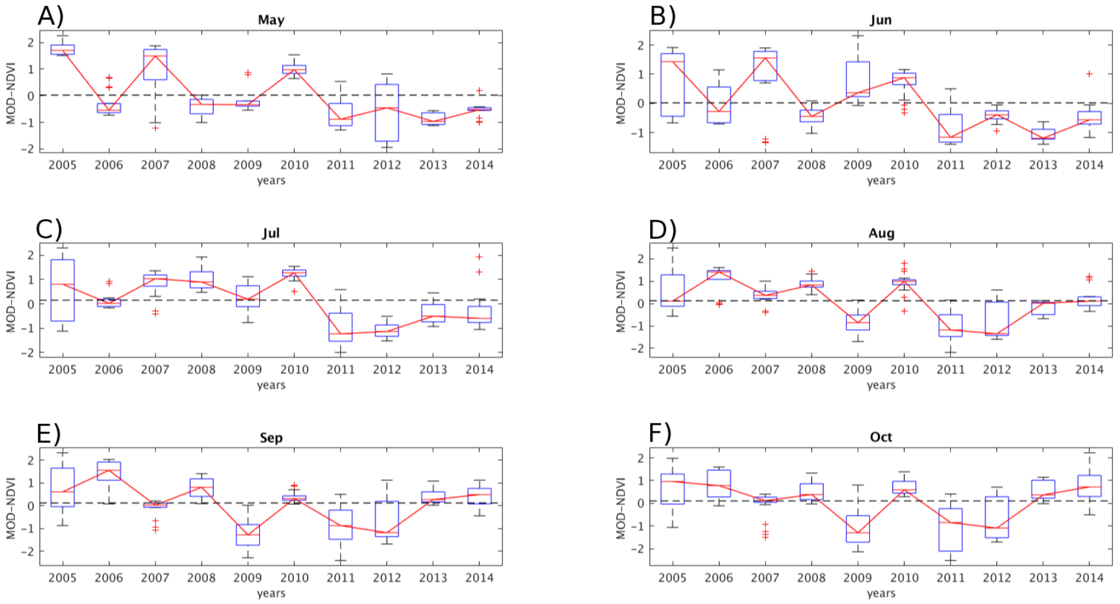

Figure S3. Box plots of Terra/MODIS NDVI standardized anomaly over the dust cluster for the years 2005–2014 between the months May–October. The panels correspond to (**A**) May; (**B**) June; (**C**) July; (**D**) August; (**E**) September; (**F**) October.

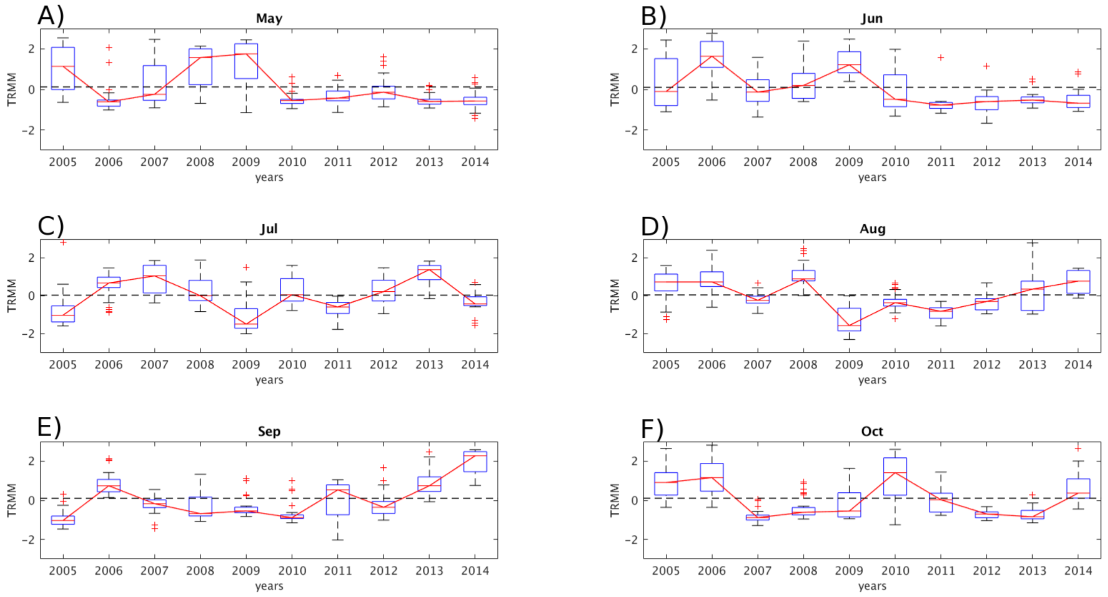

Figure S4. Box plots of TRMM rainfall rate standardized anomaly over the NAM alley for the years 2005–2014 between the months May–October. The panels correspond to (**A**) May; (**B**) June; (**C**) July; (**D**) August; (**E**)September; (**F**) October.

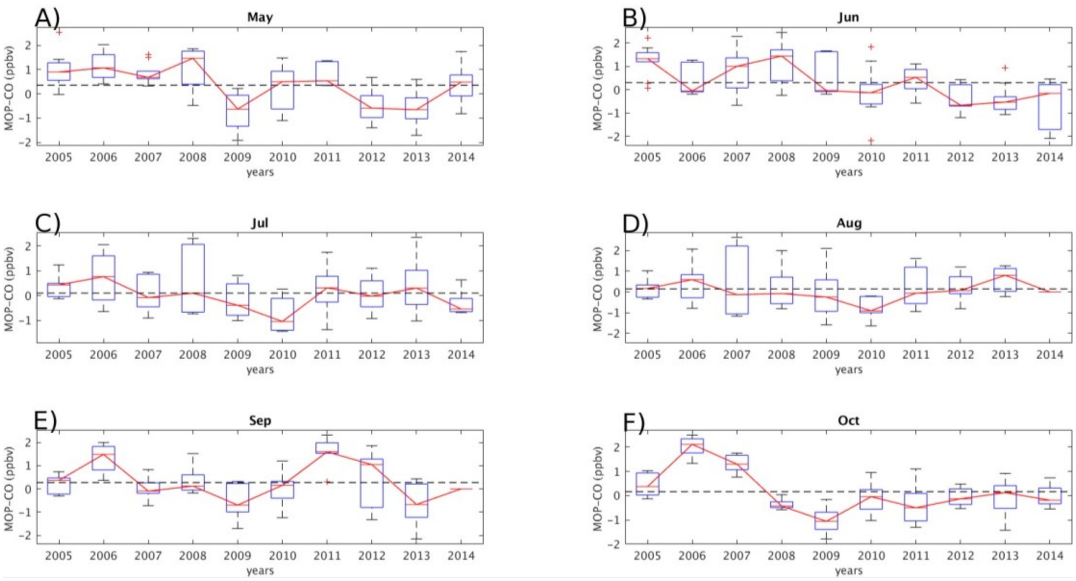

Figure S5. Box plots of MOPITT CO standardized anomaly over the anthropogenic cluster for the years 2005–2014 between the months May–October. The panels correspond to (**A**) May; (**B**) June; (**C**) July; (**D**) August; (**E**) September; (**F**) October.

© 2016 by the authors; licensee MDPI, Basel, Switzerland. This article is an open access article distributed under the terms and conditions of the Creative Commons by Attribution (CC-BY) license (http://creativecommons.org/licenses/by/4.0/).
